# The effect of computer-based cognitive flexibility training on recovery of executive function after stroke: rationale, design and methods of the TAPASS study

**DOI:** 10.1186/s12883-015-0397-y

**Published:** 2015-08-20

**Authors:** Renate M. van de Ven, Ben Schmand, Erny Groet, Dick J. Veltman, Jaap M. J. Murre

**Affiliations:** 1 Department of Psychology, University of Amsterdam, Weesperplein 4, 1018 XA Amsterdam, The Netherlands; 2 Heliomare Research and Development, Relweg 51, 1949 EC Wijk aan Zee, The Netherlands; 3 Department of Psychiatry, VU University medical center, De Boelelaan 1117, 1081 HZ Amsterdam, The Netherlands

**Keywords:** Stroke, Cognitive flexibility training, Computer-based training, Online testing, Rehabilitation outcome, Executive functions, Cognitive control, Randomized controlled trial, Cognition, Structural and functional Magnetic Resonance Imaging (MRI and fMRI)

## Abstract

**Background:**

Stroke survivors frequently suffer from executive impairments even in the chronic phase after stroke, and there is a need for improved rehabilitation of these functions. One way of improving current rehabilitation treatment may be by online cognitive training. Based on a review of the effectiveness of computer-based cognitive training in healthy elderly, we concluded that cognitive flexibility may be a key element for an effective training, which results in improvements not merely on trained tasks but also in untrained tasks (i.e., far transfer). The aim of the current study was to track the behavioral and neural effects of computer-based cognitive flexibility training after stroke. We expected that executive functioning would improve after the cognitive flexibility training, and that neural activity and connectivity would normalize towards what is seen in healthy elderly.

**Methods/design:**

The design was a multicenter, double blind, randomized controlled trial (RCT) with three groups: an experimental intervention group, an active control group who did a mock training, and a waiting list control group. Stroke patients (3 months to 5 years post-stroke) with cognitive complaints were included. Training consisted of 58 half-hour sessions spread over 12 weeks. The primary study outcome was objective executive function. Secondary measures were improvement on training tasks, cognitive flexibility, objective cognitive functioning in other domains than the executive domain, subjective cognitive and everyday life functioning, and neural correlates assessed by both structural and resting-state functional Magnetic Resonance Imaging. The three groups were compared at baseline, after six and twelve weeks of training, and four weeks after the end of the training. Furthermore, they were compared to healthy elderly who received the same training.

**Discussion:**

The cognitive flexibility training consisted of several factors deemed important for effects that go beyond improvement on merely the training task themselves. Due to the presence of two control groups, the effects of the training could be compared with spontaneous recovery and with the effects of a mock training. This study provides insight into the potential of online cognitive flexibility training after stroke. We also compared its results with the effectiveness of the same training in healthy elderly.

**Trial registration:**

The Netherlands National Trial Register NTR5174. Registered 22 May 2015.

**Electronic supplementary material:**

The online version of this article (doi:10.1186/s12883-015-0397-y) contains supplementary material, which is available to authorized users.

## Background

There is a great need for cognitive rehabilitation after stroke (i.e., brain hemorrhage or infarct). More than half of stroke patients suffer from cognitive impairment three months post-stroke [1]. Even in the chronic phase (90 days to 2 years post-stroke), approximately a third of stroke survivors still suffer from cognitive impairment [2]. Importantly, current rehabilitation programs do not seem to significantly improve executive functioning [3], whereas these impairments are related to reduced functionality in instrumental activities of daily living.

One promising way to ameliorate these impairments is to use computer games as cognitive exercises. In a recent review by our group, we concluded that cognitive training in healthy elderly subjects may result in cognitive improvement, provided that it includes frequent switching between various training tasks [4]. Such cognitive flexibility training improved cognitive functioning even in tasks that were not the focus of training, that is, the effects of the training generalized to so called ‘far transfer tasks’ [5].

Transfer of training to cognitive domains such as executive functioning, and especially generalization of training effects to the patient’s daily life functioning, is essential for clinical application of any rehabilitation technique. Moreover, effects of cognitive training may be largest in people who start at a low level of functional performance [6, 7]. Thus, it seems likely that cognitive flexibility training will result in significant improvements in cognitively impaired stroke patients. Several studies in healthy people observed changes in brain activity after intensive cognitive training, which correlated with training performance even if behavioral training effects were small [8–10]. This suggests that the training effects may leave visible traces in the brain. Against this background we planned the ‘Training Project Amsterdam Seniors and Stroke’ (TAPASS) study.

## Aim of the study

TAPASS aimed to determine whether cognitive flexibility training could improve executive functioning in stroke patients, and if so, to investigate how changes in executive functioning were correlated with functional and structural changes in the brain. We expected that cognitive flexibility training improved objective executive functioning after stroke over and above improvements due to care as usual and spontaneous recovery. More so, we expected that cognitive flexibility training would result in more executive improvement compared to an active control condition, i.e. mock training.

To determine the neural correlates of executive improvements after cognitive flexibility training we performed resting-state functional Magnetic Resonance Imaging (fMRI), Diffusion Tensor Imaging (DTI), and gray matter imaging in part of the sample. We expected that cognitive improvement would be related to changes in brain activity and structure. In particular, we expected that resting-state brain activity and structural connectivity of stroke patients who received cognitive flexibility training converged to “normal”, more so than brain activity and connectivity of those who did not receive this training.

Finally, we studied whether cognitive flexibility training was more beneficial for those with lower baseline executive performance (stroke patients compared to healthy adults), and whether this training was more beneficial in the post-acute or in the chronic phase post-stroke. We predicted that cognitive flexibility training would be more effective in stroke patients compared with healthy adults, and that it would be more effective in the post-acute phase than in the chronic phase post-stroke. We also explored which lesion characteristics (e.g., type of stroke, size and localization of lesion) and other variables (e.g., IQ, age, comorbidities, cognitive flexibility at baseline) predicted outcome.

## Design

**Fig. 1:**
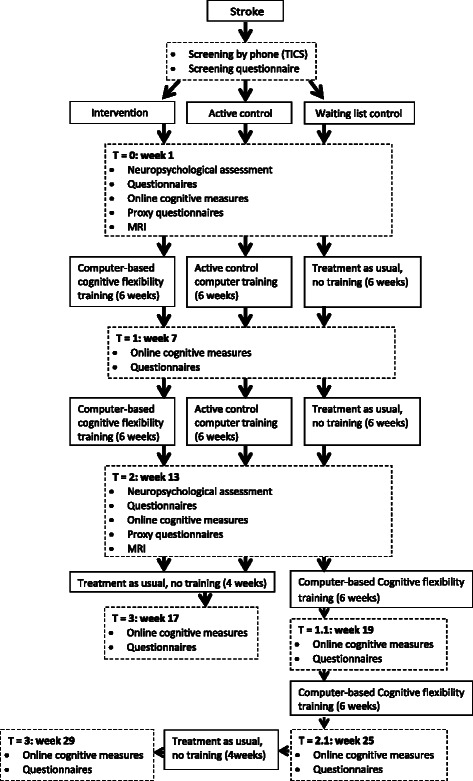
Study design flowchart of TAPASS

The design was a multicenter, double blind, randomized controlled trial (RCT) with an experimental intervention group, an active control group, and a waiting list control group. A schematic overview of the study design can be found in Fig. 1. We aimed to include 120 participants.

## Methods

### Patient sample

The TAPASS study was carried out at the University of Amsterdam. Participants were recruited from several Dutch rehabilitation centers and rehabilitation departments of hospitals. Each of the rehabilitation centers treats approximately 150 stroke patients per year, of whom at least 31–35 % have cognitive complaints [2, 11] and approximately 67 % of these also fulfill other study requirements. We expected that 35 % of the eligible group would participate in our study, which is approximately 7.5 % of the stroke patients admitted to the rehabilitation centers per year.

#### In-and exclusion criteria

The study included patients who had a stroke within the last five years, were between 30 and 80 years old, and received rehabilitation therapy as inpatient or outpatient. Participants could still be in outpatient rehabilitation treatment while living at home again. Participants needed to have cognitive impairments (not merely subjective complaints), as demonstrated by a neuropsychological assessment or as judged by a neurologist, physiatrist, psychologist, or other experienced clinician. At study entry, participants still had to have cognitive complaints. Finally, participants were required to have daily access to a computer with Internet connection and sound (either through headset or speakers), must be able to independently send emails (e.g., open emails and click on links), and able to smoothly use the mouse.

Exclusion criteria for patients were the presence of neurodegenerative disease, epilepsy, serious psychiatric illness (e.g., history of multiple psychotic episodes, acute psychosis, acute major depression), any disease other than stroke which results in severe cognitive impairments, drug or alcohol dependency, severe color blindness, severe aphasia, severe neglect, invalidating vision or hearing problems, severe computer fear disabling the participant to fully complete the neuropsychological assessment and training, and/or diagnosed learning disability (i.e., mental retardation). Furthermore, participants who were not mentally fit (defined by Telephone Interview Cognitive Status (TICS) score < 26) [12] and who were not physically fit enough (e.g., medically unstable) to be able to complete 12 weeks of training were excluded. Finally, those who were not able to understand the training instructions or who could not perform the training due to any other unforeseen reason, after instructions or after the first training week, were excluded. New participants were recruited to replace them.

Healthy elderly were recruited in another, parallel study conducted as part of TAPASS. They were elderly (older than 60 years) who did not have any cognitive complaints. The same exclusion criteria applied to the healthy elderly sample as described above for stroke patients. Also, they needed to have daily access to a computer with Internet and sound as well as basic computer skills.

For the MRI part of the study additional exclusion criteria were contraindications to MRI (see Additional file 1), such as presence of metal parts in the body and claustrophobia, and being unable to walk a small distance to reach the MRI scanner without metal support.

#### Recruitment procedures

Participants were recruited from patient databases of rehabilitation centers (Reade Amsterdam, Heliomare Wijk aan Zee, De Hoogstraat Utrecht, De Trappenberg Huizen, Adelante Hoensbroek, The Netherlands) and a hospital (Academic Medical Centre Amsterdam). Those who fulfilled the inclusion criteria were notified by their clinician, or via their caregiver, or were sent a letter with a request to participate in this study. Patients who were interested received written information regarding the study either by mail or at one of their treatment sessions. The researcher contacted the patients to provide further information (if needed) and to invite them to participate.

Additionally, participants were recruited by advertisements in newsletters and forums of Dutch stroke patients associations. The advertisement called for people who suffered from stroke less than five years ago, who (had) received rehabilitation, and who had residual cognitive complaints. Those interested in participating in our study were asked to contact the researchers, who provided them with written information about the study.

Whenever participants indicated that they wanted to participate in the study, they were asked to sign an informed consent form and complete an online screening questionnaire and a cognitive screening by phone (TICS) to assess inclusion and exclusion criteria. Permission was asked to access rehabilitation and hospital files to further establish the presence of in- and exclusion criteria (e.g., cognitive impairments during rehabilitation), inspect results of neuropsychological assessment, and to record lesion characteristics.

In total, we aimed to include at least 120 participants. Inclusion will end in March 2015, or earlier if 120 participants have been recruited. Participants received travel cost reimbursement and lifelong subscription to the training website (www.braingymmer.com).

### Intervention and control conditions

The study included an intervention group (cognitive flexibility training), an active control group (mock training), and a waiting list control group. Both training groups received 30 to 45 min of training per day, five times per week for 12 weeks. Training tasks were presented online (uva.braingymmer.com) and were done at home without the presence of a trainer. However, a trainer could be contacted via e-mail in case participants had questions or ran into problems. The trainers were trained master students who were familiar with all training tasks and login procedures. Furthermore, both training groups were contacted by phone once per week or two weeks (week 1, 2, 3, 5, 6, 8, and 10) by the trainer. Participants were given the opportunity to ask questions, and training adherence was discussed.

The tasks were designed to be visually attractive and motivating. Feedback was provided based on personal scores. After each task, feedback was given visually on a three star rating scale, with more stars for better performance. At the end of each training session participants were provided with more detailed feedback of their scores on each task.

#### Cognitive flexibility training

The intervention group received cognitive flexibility training. Nine tasks were selected to train three cognitive domains: attention, reasoning, and working memory. An elaborate description of these tasks can be found in Additional file 2. The training provided several tasks within one session, and participants were asked to frequently switch between these tasks. Each day they trained on 10 tasks for approximately three minutes each. Note that at least one of the tasks was done twice within one training session. Tasks were presented directly after each other to ensure that cognitive flexibility, that is, switching from one task to the other, was required. To become familiar with the tasks, participants trained for 10 min per task in the first week, three tasks per day. From the second week on, the number of trials per task was reduced to promote frequent switching between tasks.

The intervention training was adaptive, so that task difficulty was adapted to individual performance. Participants were instructed to go to the next level when two or three stars were obtained. However, participants could choose to stay at the same level when receiving two stars, whereas they were obliged to stay at the same level when one star or less was obtained. Level 20 was the highest level. Per day a training session with the tasks of that day was set up for each participant. The order of the tasks ensured that tasks from the same cognitive domain (attention, reasoning, and working memory) were not presented immediately after each other.

#### Mock training

The active control group received a non-adaptive mock training consisting of four computer tasks, which in our view were not likely to improve executive functioning (see Additional file 2 for a more elaborate descriptions of the tasks). The active control group switched between tasks approximately every 10 min, thus doing three tasks per day. The control tasks were presented in the same online environment as the intervention (Braingymmer). Nine levels of the tasks were selected and presented to the control group. These levels were selected to be sufficiently challenging but not too difficult. The active control group trained at one of these levels per week while the level was increased each week during the first five weeks. From week 6 to 12 the level was increased every two weeks. If, however, the participant did not master a level (i.e., did not receive at least one star), the participant was told to stay at that level until one week after they had received one star. The control training was designed to have the same amount of feedback, motivational instructions, visual stimulation, and use of mouse as the intervention training.

#### Waiting list control condition

The waiting list control group did not receive training and were not contacted by phone during the first 12 weeks. After this waiting period, all waiting list participants received the intervention training.

### Outcome measures

#### Primary outcomes

The primary outcome was objective executive functioning. Executive function was measured by five neuropsychological tasks, i.e. the number-letter switching condition corrected for the separate letter and number conditions of the Trail Making Test (TMT) from the Delis-Kaplan Executive Function System (D-Kefs) [13]; Category fluency [14]; Letter fluency [15]; Tower of London (ToL; online version based on Culbertson & Zillmer [16]); and the Wechsler adult intelligence scale Letter-Number Sequencing (WAIS III-NL) [17]. A trained master student of neuropsychology administered these tasks immediately before and immediately after the training phase (T0 and T2). The ToL was administered online without an assessor. Parallel versions were used for category and letter fluency to minimize learning effects at the second time-point. Normative data were available for the TMT, letter fluency, and for Letter- Number Sequencing. These were used to adjust for age and education differences.

For category fluency, participants had to mention as many words as possible of a given category within one minute. As most participants had already performed the commonly used versions of the fluency task (animals, occupations), we used four different categories. Either male names and supermarket articles, or female names and city names were used as categories. The versions used were counterbalanced over participants such that at follow-up they did the other version.

We added a switch condition to the category fluency task. After naming words of category one, participants named words from category two, and finally they were asked to alternate between mentioning a word from category one with a word from category two (i.e., the switch condition). In all fluency conditions, participants had to mention as many words as possible within one minute. Scores for the single condition were the numbers of correct words mentioned within one minute. For the switch condition, the score was calculated by subtracting the total number of correct words in the switch condition from the average of the two single conditions (i.e., switch cost = (category 1 + category 2)/2 - switch category). Higher switch costs reflect lower cognitive flexibility.

#### Secondary outcomes

##### Switch dual task

Cognitive functions related to the training, in particular cognitive flexibility, were measured by switch cost and dual cost task. These measures were derived from a combined version of a switch task [18] and a dual task [19]. These switch and dual tasks were combined to reduce testing time. The switch and dual tasks together take approximately 30 min to administer. Furthermore, the original instructions of the switch task (i.e., to differentiate between vowels and consonants, and between odd and even digits) appeared to be too difficult in a pilot study in healthy elderly and stroke patients. Thus, a modified version was used. The details of the combined and modified version of the switch and dual task can be found in Additional file 3.

##### Training outcome

Improvement on training tasks was registered automatically by the training website. Performance was evaluated in the intervention group by the level the participant had reached and the number of stars he or she obtained at each level. Note that the active control group trained on a predefined level. In this group, performance was based only on the number of stars obtained at each of these predefined levels. Additionally, each time the participant completed a task, he or she obtained a score on that task. For each task, a summation of all scores obtained was registered as a cumulative score. Thus, one cumulative score existed for each task that was done by the participant. In both groups, a global performance estimate was based on the z-scores of the cumulative scores of each performed training task. To calculate these z-scores, the mean and standard deviation per task of the final cumulative score at the end of the training of the whole sample was used.

##### Objective cognitive improvement

To determine the far transfer effects of the training, objective cognitive improvement was measured using 12 neuropsychological and computer tasks (see Table 1). To reduce the number of dependent variables, composite scores were calculated based on standard scores of the following cognitive domains: attention, memory, working memory, reasoning, and psychomotor speed. Tasks used to compute these composite domain scores can be found in Table 1. Scores were expressed as demographically corrected z-scores, where possible. If normative data for a task were not available, demographically uncorrected z-scores for all time-points were calculated based on the standard deviation and mean of the sample at baseline. In Additional file 4 it can be found when these measures were administered and in Additional file 5 a description of several computer tasks is given.

**Table 1:** Composite scores of several cognitive domains

Cognitive domain	Task
Primary outcome measures	
Executive functioning	- D-Kefs TMT number-letter switching condition corrected for the separate letter and number conditions [13]
	- Category fluency [14]
	- Letter fluency [13, 15]
	- Tower of London^a^ [20]
	- Wechsler adult intelligence scale Letter-Number Sequencing [17]
Secondary outcome measures	
Attention	- Trail Making Test contrast condition B corrected for A^b^ (TMT; NeuroTask BV)
	- Paced Auditory Serial Addition Task (PASAT) [21]
	- Digit-Symbol-Coding^a^ (DSC) [17]
Memory	- Rey’s auditory verbal learning test (RAVLT) [22]
Working Memory	- Operation span^ab^
	- N-back^b^ [23]
	- Blokkenreeksen (NeuroTask BV); online modified version of Corsi task^ab^
Reasoning	- Raven Coloured Progressive Matrices^a^ (CPM) [24]
	- Shipley Institute of Living Scale-2^a^ [25]
Psychomotor speed	- D-Kefs TMT motor speed condition [13]
	- Mouse skills tasks^ab^ (NeuroTask BV)
Inhibition	- stop-signal task^b^

Note. D-Kefs TMT = Delis-Kaplan Executive Function System Trail Making Test

^a^ = Online measures; ^b^ = See Additional file 5 for a description of this task

##### Subjective cognitive functioning

Subjective cognitive functioning was assessed by five online questionnaires. A proxy of the participant also completed some of these questionnaires. Subjective executive functioning was measured by the Dysexecutive Questionnaire (DEX; participant and proxy) [26], cognitive complaints by the Cognitive Failure Questionnaire (CFQ; participant and proxy) [27], participation in everyday life activities by the modified version of the Utrechtse Schaal voor Evaluatie en Revalidatie- Participatie (USER-P; participant) [28], instrumental activities of daily living by the Lawton & Brody Instrumental activity of daily living scale (IADL; participant and proxy) [29], and quality of life by the Short Form Health Survey (SF-36; participant) [30]. These questionnaires are brief and are commonly used in clinical and research settings. See Additional file 4 for administration occasions.

##### MRI

Secondary outcome measures included MRI. In all MRI analyses, a mask of the lesion was created to ensure that this part would not be taken into account during analysis. Analyses consisted of resting-state fMRI, DTI, and voxel based morphometry (VBM) data. The results of these analyses were compared with those of healthy elderly (which were obtained in a parallel study conducted in the TAPASS project).

Functional connectivity was assessed using resting state fMRI. Resting-state fMRI data were analyzed with independent component analyses (ICA) and graph theoretical metrics such as degree distribution as a measure of resilience or robustness [31]. This measure reflects ‘the distribution of the number of connections linking each node to other nodes throughout the network’ [31]. Furthermore, global and local efficiency of information exchange, small-worldness, and centrality were quantified. Cardiac and respiratory function, and a questionnaire determining what participants were thinking during the resting-state scanning (measured by the Amsterdam Resting-State Questionnaire; ARSQ) [32], were obtained as potentially confounding factors. Furthermore, participants rated whether they had fallen asleep during resting-state fMRI.

Structural connectivity was assessed based on white matter tracts analyses. White matter was measured with diffusion weighted imaging. DTI measures were used to assess global and local structural connectivity. Gray matter was measured with T1 weighted structural MRI scans. VBM was used to examine possible gray matter changes due to training, with a focus on the frontal lobe. Furthermore, lesion characteristics such as type of stroke, lesion size and location, diffuse versus local damage, and size of the damaged network, were derived from FLAIR and T2 weighted structural MRI scans to examine whether they could predict training outcome.

#### Other parameters

##### Compliance, blinding, and other training-related measures

During the training the following data were registered by the training website or researcher: amount of training sessions actually performed, amount of extra personal contact (phone or email) due to questions or technical issues during training, and level of engagement (i.e., how often a reminder to train was needed). Furthermore, participants recorded in a daily log their level of motivation during training, amount of physical exercise at the day of training, how interesting and difficult were the tasks of that day, and fatigue level before and after training. Once every week or every two weeks, participants were asked by phone (see procedures section) about the amount of cognitive or physical rehabilitation received during the past period. Finally, after six weeks of training, at the end of training, and at the end of the study, an exit questionnaire was administered including questions about subjective training effectiveness; change of strategies during training; check of blinding to experimental condition; changes in cognitive stimulation in daily life besides study related training; and major changes during training (e.g., change of medication or major life event).

##### Clinical measures

Stroke measures were obtained at baseline. These were age at stroke, time since stroke, cognitive complaints immediately after stroke, received rehabilitation prior to the study, and work status. Whenever possible this information was checked in the medical files. Furthermore, the participants rated their recovery after stroke on a 10 cm vertical visual analogue scale.

Depression was assessed with the Hospital Anxiety Depression Scale (HADS) [33]. Fatigue was assessed with the Checklist Individual Strength- Fatigue subscale (CIS-f) [34]. Prior to training, cognitive status was screened with the TICS [35] Dutch version of Brandt, Spencer, Folstein (1988). Furthermore, prior to training, visuoperception was measured with D-KEFS visual scanning condition [13] to ensure absence of severe neglect.

Several possible confounding factors were registered. Educational level was estimated based on UNESCO ISCED 1997. Comorbidity was assessed by asking the participants whether they were under medical treatment for anything else than stroke. Moreover, participants were asked about their alcohol and drugs use, occupation, prior neuropsychological assessment, participation in similar research projects, mouse skills and computer aversion, and previous computer game experience.

#### Sample size

We were interested in large differences between the treatment and control groups. A difference of one standard deviation was considered clinically significant. Therefore, assuming a power of .80 and alpha of .05 (one tailed), an effect size of 1 standard deviation would be detected with a sample size of 28 (2 × 14). A large effect size (*d* = 0.80) would be detected at a sample size of 40 (2 × 20).

With a sample size of 120 (3 × 40) participants and with a power of .80 and alpha of .05 (one tailed), an effect size of *d* = 0.56 (medium) could be detected. Karbach and Kray [5] found an average effect size for far transfer effects of *d* = 0.40. This effect size was already found after four training sessions. Therefore, it was expected that in our training, with 58 sessions, the effect size would be at least *d* = 0.56. Furthermore, Westerberg et al. [36] found an effect size of *d* = 0.80 for subjective cognitive complaints (measured by the CFQ) after 16.6 h of working memory training. This effect would be revealed with 40 (2 × 20) participants. Attrition was expected to be approximately 15 %. Therefore, we expected that 138 participants had to be included to reach a sample size of 120.

In all conditions, a subset of participants was scanned. Thus far, it is not known what the effect sizes are of the MRI outcome measures. Earlier studies that were able to reveal neural changes related to training included 11–33 participants per condition [9, 10, 37, 38]. However, these studies have been conducted in healthy subjects who probably have less variability in brain anatomy and function. It was nevertheless expected that a sample size of 20 per condition should be sufficient to demonstrate neural alterations related to intensive cognitive training. At follow-up, we expected to be able to reexamine at least 65 % of the participants who were scanned at T0. Therefore, we expected to be able to analyze 13 participants per condition, which should have been sufficient for the planned analyses.

### Procedures

#### Randomization and blinding

Participants were randomized evenly into three groups, that is, the intervention group (cognitive flexibility training), the active control group (mock training), or the waiting list control group. This was done as soon as the telephone screening (TICS) had been administered, thus, before medical files had been received. A minimization technique [39], implemented in the software of Minimpy, was used to minimize imbalance between the conditions for time since stroke (post-acute versus chronic), level of computer experience, age, scores on TICS, and sex.

In case the medical file revealed that a participant did not fulfill in- or exclusion criteria, he or she was contacted to notify them about their exclusion and was replaced by a new participant. Excluded waiting list participants who already started the waiting period were given access to the training immediately. No further measurements were performed in those participants.

Both the participants and the assessors of neuropsychological tasks were kept blind to the training conditions, and the assessor was also kept blind to who was in the waiting list condition. Participants were told that the study aimed to compare two types of cognitive training by computer tasks, but not that one training was expected to result in superior improvements. Moreover, they were asked not to talk about training content during follow-up assessments to ensure that assessors remained blind to training allocation. Likewise, they were asked not to talk about the training sessions with other study participants. Note that assessors of computer tasks were not blinded to condition as they provided the training instructions.

#### Training instructions

Training instructions were given at baseline (T0) for the training groups or after the waiting period (T2) for the waiting list group. A trained master student at the University of Amsterdam gave these instructions. If the participant preferred, a different location could be selected. During an instruction session, instruction videos were shown of each training task on individual computers, after which the participant practiced the task. A researcher was present to explain the training and to answer questions. An instruction booklet was provided that also included the instructions.

Participants were asked to train at moments when they knew they had at least 50 min available so that they would not be under time pressure. Moreover, they were asked to train at moments of the day when they were not mentally fatigued (e.g., not to train late at night). Participants were stimulated to perform well. Both training groups were provided with the same motivational instructions. Furthermore, they were informed that their training frequency was monitored. Since performance on training tasks would be analyzed afterwards, it was emphasized that only the participant himself or herself should use the personal login codes. Thus, only the participant should do the tasks on his/her own account. The use of brain training programs, other than the study training, during study period was not allowed. Importantly, any other treatment that was ongoing at enrollment in the study was continued (care as usual).

During the training, participants kept a daily log to register subjective performance on the training tasks, motivation and fatigue during training, amount of physical activity at the day of the training, and potential technical issues of the training. The log took less than five minutes to complete. Participants received a reminder e-mail when they did not train for two days and were contacted by the researcher after three days without training.

#### Data acquisition

Prior to the training or waiting period (T0) and at the end of the training or waiting period (T2) neuropsychological tasks were administered to measure far transfer effects of the training. Some of the measurements were performed online. Furthermore, five questionnaires were administered online at these time-points to determine health status, participation in daily life activities, and subjective cognitive functioning. The proxy of the participant also completed three of these questionnaires online. Participants were asked to do all online measurements of the different time-points on the same computer. A global outline of all measurement time-points can be found in Table 2.

**Table 2:** Schematic outline of measurements per time-point

	T0	T1	T2	T1.1	T2.1	T3
	Week 1	Week 7	Week 13	Week 19^a^	Week 25^a^	Week 17^b^/ 29^a^
Neuropsychological assessment	X		X			
Questionnaires	X	X	X	X^a^	X^a^	X
Online cognitive measures	X	X	X	X^a^	X^a^	X
Proxy questionnaires	X		X			
MRI	X^c^		X^c^			

Note. X = administered at time-point. A detailed overview can be found in Additional file 4

^a^Only done by waiting list group; ^b^Only done by intervention and active control group, ^c^only done by subgroup of study sample

The tasks and questionnaires were spread over three days (two days at home and one day at the University of Amsterdam or an alternative location). The tasks done at home were performed online without assessor (see Additional file 4). The remaining tasks were done with an assessor at the University of Amsterdam or alternative location. Importantly, some neuropsychological tasks were administered both online (without assessor) and as paper and pencil tasks (with assessor). The order of these tasks was counterbalanced over days such that either the computer tasks were done on day one and the paper and pencil tasks on day two, or vice versa.

In order to compare our study with other training studies, which mostly consisted of six weeks of training or less [36, 40, 41], a subset of online tasks and questionnaires were administered six weeks after training onset (T1; see Additional file 4). These tasks were done at home instead of two training sessions.

After 6 and 12 weeks of training, a brief questionnaire regarding subjective training success was administered. Finally, to measure long-term effects of the training, a subset of online tasks and questionnaires were administered four weeks after training completion (T3; Additional file 4). Note that the switch task was administered online four times (T0, T1, T2, and T3). The dual task was administered at T0 and T2 only. An assessor was present at T0 and T2 when the switch and dual task was done at the University, but not at T1 and T3 when the switch task was done at home. An overview of tasks administered at different time-points can be found in Additional file 4.

The waiting list control group received the same measurements as the training groups. They did not, however, receive any training during the first 13 weeks. Training commenced immediately after the 12 weeks waiting period measurements (T2). The same tasks performed after six weeks of waiting (T1) were repeated after six weeks of training (T1.1) and at the end of the training (T2.1, see Table 2). Finally, similar to the training groups, there was a follow-up measurement four weeks after training completion (T3). Note that to minimize extra effort, the waiting list control group only performed the online measures after training completion and did not visit the University of Amsterdam again.

##### MRI

Before and after the training or waiting list period, a selected subset of participants was scanned. They were participants who were able to come to the MRI scanner and who fulfilled all MRI inclusion and exclusion criteria. Participants were screened with an MRI safety checklist. Because we used a resting-state protocol, participants were not required to perform any tasks during the scans. However, one questionnaire was administered within the MRI scanner immediately after the resting-state scan to assess their mental state during that scan. Further MRI procedures, including the scan sequences used, can be found in Additional file 1.

The current study was approved by the Medical Ethical Review Board of the VU University Medical Center, Amsterdam. TAPASS is registered with the Central Committee on Research Involving Human Subjects Register NL4468502913 (www.toetsingonline.nl). Inclusion started in September 2013.

### Statistical analyses

P-values of .05 or lower (one tailed) were considered significant. Sociodemographics (age and education) and clinical data (time since stroke) at baseline were evaluated with Student *t*-test or Mann–Whitney *U*-test and were added as covariates. Additionally, number of training sessions completed was added as a covariate.

To evaluate blinding success, the two trainings were compared to check if the mock training was not perceived as a control training. Reported motivation, training compliance, and check of blinding were analyzed with Mann–Whitney *U*-test or *χ*^2^.

Primary analyses were performed with mixed model analyses. The dependent variables were switch cost on the DKEFS TMT, number of words mentioned during the category fluency and the letter fluency task, number of steps and execution time of the ToL, and score on Letter-Number Sequencing. The independent variable was condition (intervention, active control training, and waiting list control group). Time-points in this model were before and after training.

Secondary analyses were performed in a similar way. However, the dependent variables were cognitive flexibility based on switch cost and dual cost; objective cognitive function based on the composite scores of attention, memory, working memory, reasoning, inhibition, and psychomotor speed; training improvement; and subjective cognitive and everyday life functioning.

Whenever intervention resulted in a significant improvement of the dependent variables that were additionally measured at T1 (after six weeks of training) and T3 (follow-up four weeks after training completion), the time-points T1 and T3 were added to the model. This was done to determine whether the training was already effective after six weeks of training and to establish whether training effects would persist after the training.

MRI analysis for the resting-state data were performed with ICA and dual regression and graph analysis. Cardiac and respiratory function, time since stroke, and scores on the ARSQ were treated as potentially confounding factors. DTI data were analyzed using tract-based spatial statistics [42]. Gray matter was analyzed with VBM [43]. Mixed ANOVA’s were used to analyze differences in changes from baseline after training between intervention and active control group of the values derived from DTI analyses, VBM values, and graph metrics.

Finally, we explored whether the effect of cognitive flexibility training varied as a function of baseline executive functioning, health state (stroke versus healthy) and time since stroke (sub-acute versus chronic). Linear regression was used to determine predictors of training outcome.

All analyses were performed as intention-to-treat analysis, including all participants who started the training. Additionally, analyses were rerun as per-protocol analysis. In these analyses, only the participants who completed the training according to the protocol were included in the analyses.

## Discussion

Our study is one of the first to test the effect of a computer-based training aimed at improvement of executive functioning in stroke patients. Innovative features of the study are that cognitive flexibility is trained specifically, that it is being done via the Internet, and that the study is a double-blind RCT.

Several earlier studies have reported positive transfer effects of computer-based cognitive training in healthy elderly [38, 44, 45], but far transfer effects on tasks dissimilar to the training are not always found [9, 46]. Beneficial effects have also been reported in stroke or other acquired brain injury populations [6, 36, 41]. It remains, however, uncertain under what conditions computer-based training is effective.

The current training has not been proven effective yet in the healthy population or in stroke survivors. Nevertheless, it consisted of several factors that are likely important to induce far transfer effects [4]. Most importantly, the training included a switch element. The training did not only train one specific cognitive domain, but it also trained cognitive flexibility, which is a higher-level cognitive function, called upon in many tasks. As a result, it is likely that the training would lead to improvements in other, untrained tasks that do require cognitive flexibility.

Length of the training also seems to be important. Basak, Boot, Voss, and Kramer [44] found a transfer effect of their video game training of executive functions after 23.5 h of training, but not yet after 11 h of training. The current training contained 58 sessions of half an hour, adding up to a total of 29 h.

Another important factor for far transfer effects of training is that training should be adaptive [47]. Difficulty level of the intervention training was adapted to the performance of the participant. This was done after completion of one round of a training task.

Training may have a larger effect on everyday life functioning in stroke patients than in the healthy population. People who have suffered from stroke commonly have lower baseline cognitive functioning than healthy elderly. There is more room for improvement compared to healthy elderly [6, 7]. Also, stroke patients may be more motivated to improve their functioning precisely because they have lost much of their function.

There are several potential pitfalls in the current study. As the training and several outcome measure tasks were done online on the participant’s own computer, computers may have had different settings (e.g., different screen sizes and, therefore, different size of training or tasks presentation). These settings could affect outcome measures. To minimize the effect of setting differences, participants were asked to do all measurements of the different time-points on the same computer. Furthermore, due to continuous changes within Internet browsers (e.g., changes of packages used for flash tasks), technical failures could have occurred. To reduce the risk of technical failures, the participants were advised to install comparatively stable Internet browsers (e.g., Mozilla Firefox or Safari). All instructions for installing browsers, starting the training, doing the online tasks etc. were thoroughly explained face to face and in an instruction booklet.

Motivation and fatigue of the participants possibly played a major role in this study. Participants were required to spend approximately 50 h on the TAPASS project. This is a large investment, which may have led to fatigue and loss of motivation. In turn, this may have negatively influenced the outcome measures. To avoid loss of motivation, participants were contacted by phone once a week or every two weeks. Positive feedback was given after completion of each training session. The assessment of the outcome measures was spread over three days, to reduce the influences of fatigue. Extra breaks were offered when needed.

One of the major strengths of this study is the design with two control groups. Most previous training studies either did not include a control group or provided a less motivating training as control condition [47]. This study included both an active control group and a waiting list control group. The active control group received a mock training we considered was motivating and visually stimulating. We checked this assumption in the exit questionnaire. This is important not only to keep participants motivated to train for 12 weeks, but also to ensure that participants remained blind to training condition. As the mock training could still have been quite challenging, this training may also have improved cognitive functioning. It was, however, not expected that improvement would transfer to tasks dissimilar to the training. In case both trainings turned out to be effective, the effects of spontaneous recovery and learning could be taken into account based on comparisons with the waiting list control group.

The training could be done at home and most assessments were also in-home. A large sample could, consequently, be acquired and a follow-up measurement could be done after four weeks. The large sample size is another strength of this study. In case of a null effect, the results would still provide valuable insights, as statistical power was sufficient to detect medium-sized effects. The results of this study were compared with the results of a parallel study of computer-based cognitive flexibility training in healthy elderly. We expected that those who have suffered from stroke and currently have cognitive complaints will have a lower cognitive baseline than healthy elderly. This study, thus, gives insight into the effect of different baseline cognitive abilities on training outcome.

## Conclusions

There is a need for improvement of rehabilitation of executive functions in the stroke population. In case this training proves effective, the results of this study will provide an important contribution to the rehabilitation of executive impairments after stroke. This study additionally provides insights into the field of cognitive training as it not only looks at behavioral effects, but also at changes in the brain.

## Additional files

Additional file 1: **MRI Scan protocol.** (PDF 273 kb)
Additional file 2: **Description of training tasks.** (PDF 712 kb)
Additional file 3: **Details of the switch and dual task.** (PDF 389 kb)
Additional file 4: **Measures at different time-points.** (PDF 370 kb)
Additional file 5: **Description of the computer tasks.** [48–52] (PDF 529 kb)

## Abbreviation

ARSQ: Amsterdam Resting-State Questionnaire
CFQ: Cognitive Failure Questionnaire
CIS-F: Checklist Individual Strength- Fatigue subscale
CPM: Raven Coloured Progressive Matrices
DEX: Dysexecutive Questionnaire
D-Kefs TMT: Delis- Kaplan Executive Function System Trail Making Test
DSC: Digit-Symbol-Coding
DSST: Digit Symbol substitution Task
DTI: Diffusion Tensor Imaging
fMRI: Functional Magnetic Resonance Imaging
HADS: Hospital Anxiety Depression Scale
IADL: Instrumental activity of daily life scale
ICA: Independent component analyses
MRI: Magnetic Resonance Imaging
O-span: Operation span
PASAT: Paced Auditory Serial Addition Test
RAVLT: Rey Auditory Verbal Learning Task
RCT: Randomized controlled trial
SF-36: Short Form Health Survey
TAPASS: Training Project Amsterdam Seniors and Stroke
TICS: Telephone Interview Cognitive Status
TMT: Trail Making Test
ToL: Tower of London
USER-P: Utrechtse Schaal voor Evaluatie en Revalidatie- Participatie
VBM: Voxel based morphometry
