# Supplementary material for: The effect of computer-based cognitive flexibility training on recovery of executive function after stroke: rationale, design and methods of the TAPASS study
Source: BMC Neurol. 2015 Aug 20;15:144. doi: 10.1186/s12883-015-0397-y (PMC4545547; doi:10.1186/s12883-015-0397-y)
Supplement: Additional file 1: — MRI Scan protocol. (PDF 273 kb) [file 12883_2015_397_MOESM1_ESM.pdf]

Additional file 1

MRI Scan protocol

Participants were scanned in the 3T MRI scanner of the Spinoza Centre in Amsterdam. First, instructions about the scanner were given in a room containing a mock scanner. The importance of not moving during the scan session was stressed. Participants were screened for contraindications of MRI scanning (e.g., presence of metal or neuro-stimulator). After the screening, the instructions of the resting-state scan and the way of responding to the ARSQ were practiced. The latter was done because no verbal instructions could be given between the resting-state fMRI scan and the questionnaire to avoid interference with their thoughts.

The ARSQ consists of 30 questions about feelings, sensations, and thoughts. Questions could be answered by moving a selection box either to left or to the right. The five response alternatives ranged from definitely disagree to definitely agree. An example of a question was “I thought about others”.

The total scan protocol within the MRI scanner took approximately one hour. The sequences used can be found in table A1. During most of the scanning, the participants did not need to do anything else than to lie still. A movie was presented on a screen to keep participants busy. They could watch the movie or they could fall asleep. Before the resting-state scan, participants aurally and visually received the following instructions in Dutch: ”The following part will take 10 minutes. It is important to relax, keep your eyes open and look at the cross on the screen. You are allowed to blink. Unleash your thoughts and don’t dwell on one thought for too long. Try not to move and not to fall asleep.”

During the resting state scan of 10 minutes, a fixation cross was presented at the middle of the screen. After the scan, participants were shown the following instructions on the screen: *“*Now several statements will follow regarding potential feelings and thoughts you may have experienced during the resting period. Please indicate the extent to which you agree with each statement” [32]. Participants answered the questions while lying in the scanner using three buttons of a button box. After completing the questionnaire, participants were asked whether they had fallen asleep during the resting-state scan.

Table A1

MRI sequence protocol including duration of scans and what is presented on the screen

Scan	Duration in minutes	Presented on screen
1) **SmartBrain**	1	Movie
2) **RefScan4**	1	Movie
3) **T1** (sT13DTFE_P25_S2_6m_SENSE)	6	Movie
4) **B0**	2	Movie
5) **Resting-state EPI** (TRA_3mm_ISO)	10	Instruction followed by cross on screen
**ARSQ**		Questionnaire + "Did you fall asleep"
6) **T2** (WIP_3D_Brain_View_T2_32chSHC)	5	Movie
7) **FLAIR** (WIP_3D_Brain_View_FLAIR_SHC_SENSE)	4	Movie
8) **RefScan4**	1	Movie
**9) DWI** (DTI32_Sense2(PA))	5	Movie
**10) DWI** (DTI32_Sense2(AP))	5	Movie
**11) T1** (sT13DTFE_P25_S2_6m_SENSE)	6	Movie
