# Supplementary material for: The effect of computer-based cognitive flexibility training on recovery of executive function after stroke: rationale, design and methods of the TAPASS study
Source: BMC Neurol. 2015 Aug 20;15:144. doi: 10.1186/s12883-015-0397-y (PMC4545547; doi:10.1186/s12883-015-0397-y)
Supplement: Additional file 2: — Description of training tasks. (PDF 712 kb) [file 12883_2015_397_MOESM2_ESM.pdf]

Additional file 2
Description of training tasks

The training can be reviewed on www.braingymmer.nl

Cognitive flexibility training
All tasks consist of 20 levels.

Attention tasks

Pattern Matrix

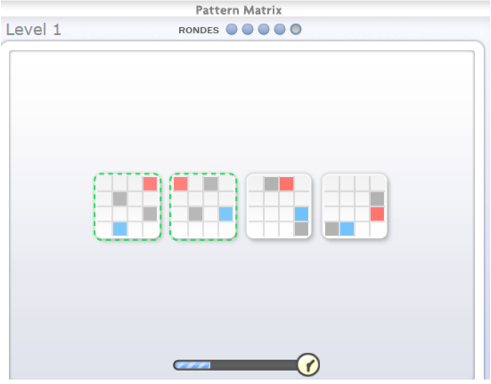

Participants have to mentally rotate patterns to find pairs of equal patterns. This task is done under time pressure. The higher the level, the more difficult the patterns become and more than four patterns are presented.

Birds of a Feather

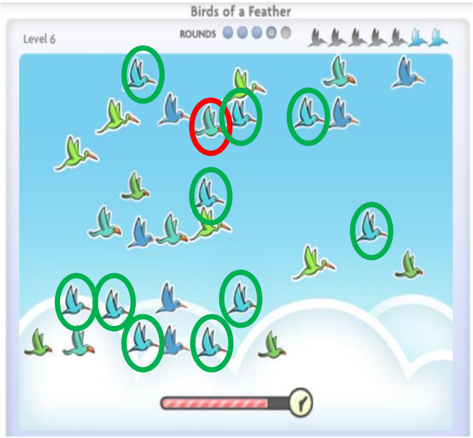

Participants have to count particular light blue birds under time pressure. The higher the level, the more similar the distractor and the to be counted birds become.

Mind the Mole

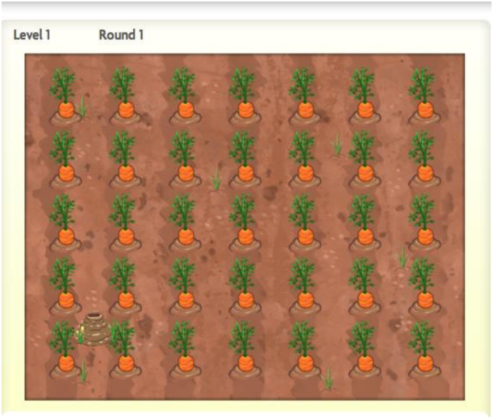

Moles appear on the screen and make a movement in a certain direction. The participant has to click on a mole as soon as it starts to move in a different direction.

Reasoning tasks

Square Logic

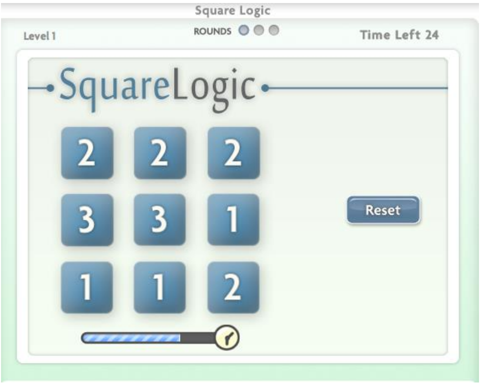

Blocks have to be placed on top of each other (the covered block will disappear) such that only one block will remain. Blocks can only be placed on adjacent blocks and are exactly one number higher or lower. This task is done under time pressure. Higher levels include more blocks and more numbers.

Out of Order

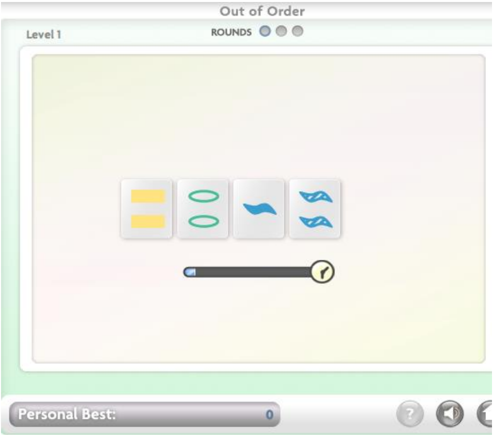

Tiles need to be arranged in such a way that they match with their neighbor tile at least on one characteristic. Characteristics are color, shape, filling, and number of figures. This task is under time pressure and has more tiles at higher levels.

Patterned Logic

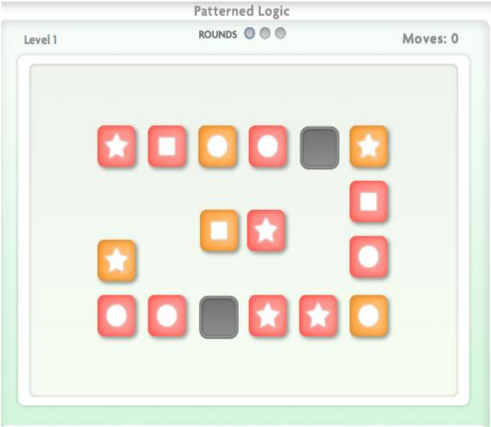

A series of tiles is shown with a color pattern and a figure pattern. Participants have to complete the missing tiles in the series based on one of these two patterns. The tiles to be placed are in the center.

Higher level series have more gaps.

Memory tasks

Toy Shop

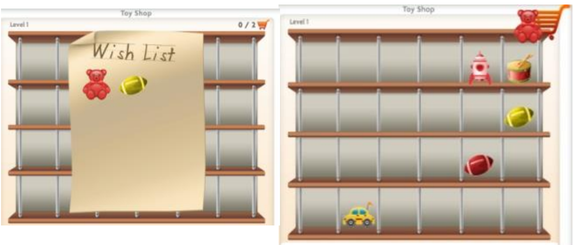

Participants have to remember items from a shopping list and collect these items from a store.

Multi Memory

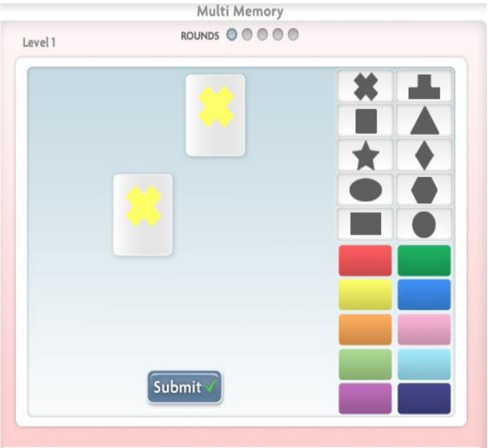

Several figures are shown in different colors and shapes. The participants have to reconstruct these figures after they have disappeared.
The number of figures to remember/reconstruct gradually increases at higher levels.

Moving Memory

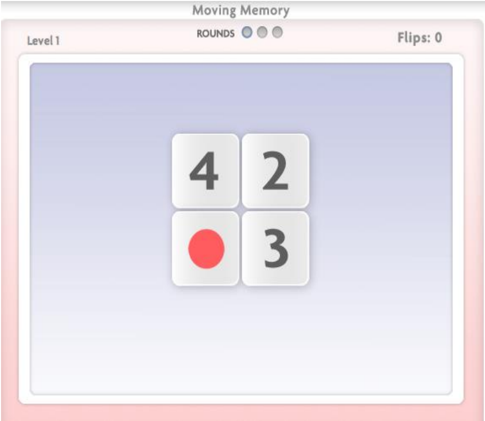

Participants have to find pairs of matching shapes, which are on the reverse side of each uniquely numbered tile. However, tiles change position after a pair has been found. Thus, shapes can only be remembered based on the numbers on the tiles and location plus movement. Higher levels have more pairs.

Active control training
All tasks consist of nine levels.

Fuzzle

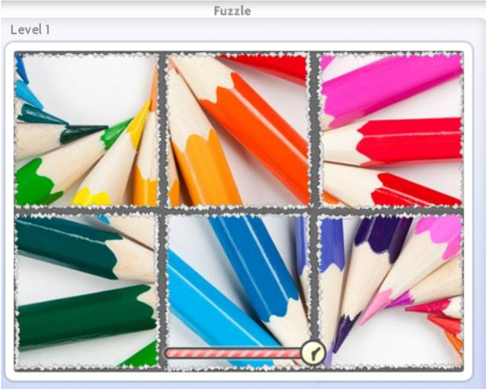

Participants have to reconstruct a fractured picture. Pictures are fractured in more pieces at higher levels.

Sliding Search

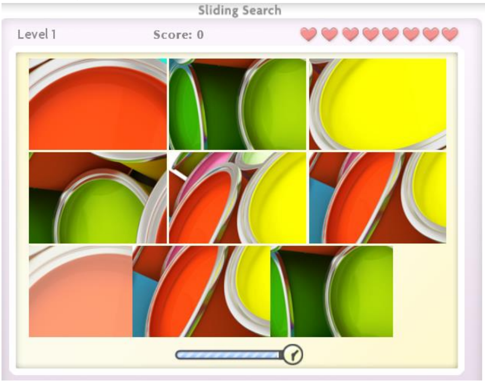

An exact copy of one of the six pictures presented in the top of the screen passes by at the lower part of the screen. Participants need to match pictures from the upper part of the screen with those in the lower part of the screen by dragging the picture from the top to the exact copy in the bottom. Pictures pass by faster at higher levels.

Pay attention

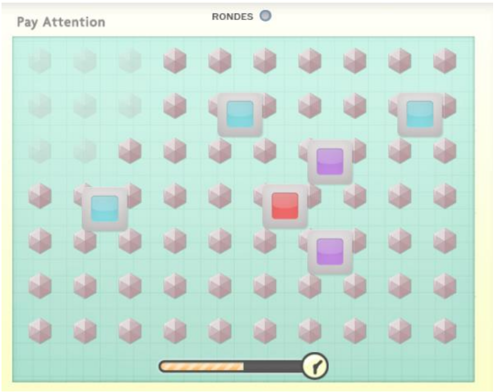

Squares appear on a screen. Whenever they change color, the participant has to click them. Higher levels include more squares.

Grid Tracks

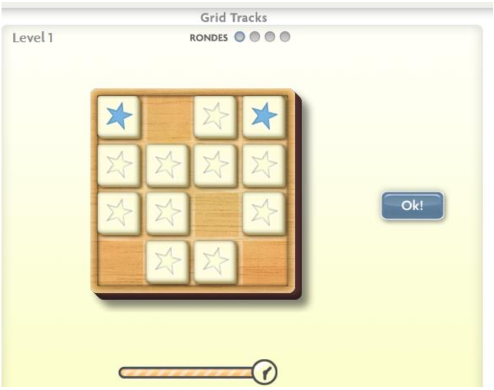

Participants have to mentally follow the trajectory of a couple of target blocks, which initially have blue stars. When blocks stop moving, they need to locate and click on the target blocks. Higher levels include more target blocks.
