# Supplementary material for: The effect of computer-based cognitive flexibility training on recovery of executive function after stroke: rationale, design and methods of the TAPASS study
Source: BMC Neurol. 2015 Aug 20;15:144. doi: 10.1186/s12883-015-0397-y (PMC4545547; doi:10.1186/s12883-015-0397-y)
Supplement: Additional file 3: — Details of the switch and dual task. (PDF 389 kb) [file 12883_2015_397_MOESM3_ESM.pdf]

Additional file 3

Details of the switch and dual task

In the switch and dual task a letter and a digit were presented in one of the four quadrants of a square (approximately 5 x 5 centimeters; font Courier New with size 16px; see figure A1). Participants had to either respond to the letter or to the digit. The digit task was to decide whether a digit was either higher or lower than five. The digit '5' itself was never presented. The button 'v' had to be pressed for a digit lower than five; the 'n' had to be pressed for higher than five. Similarly, the letter task was to decide whether the letter was uppercase or lowercase. The button 'v' had to be pressed for the lowercase letters 'e d h t'. The button 'n' had to be pressed for the uppercase letters 'R B A F'. Thus, correct button presses were left: v = lowercase letter and lower than five; right: n= uppercase letter and higher than five.

The instruction of the task depended on which quadrant the stimuli were presented in. There were two versions of the task to avoid effects of eye movements. In the horizontal version, the letter task had to be done whenever the stimulus appeared in one of the top quadrants, and the digit task had to be done when it appeared in one of the bottom quadrants. In the vertical version, the letter task had to be done when the stimulus appeared in one of the two right quadrants, and the digit task had to be done when it appeared in the left quadrants. The task version was counterbalanced over participants, but each participant received the same version at all measurement occasions.

During the switch task, the stimuli were predictively presented in one of the four quadrants in a clockwise order. Thus, participant started with two letter trials and switched to the digit task after two trials, etcetera (i.e., AABBAABB etc. see figure A1a). Participants first practiced the two tasks separately in two blocks of 24 trials per task. Afterwards, the two tasks were combined and were practiced in 32 trials. The actual task consisted of four blocks of 48 trials. The first four trials of each block were discarded from analysis. This was done to ensure that participants were in the task flow. Switch cost was calculated by subtracting the reaction time and accuracy of the non-switch trials from the switch trials. The larger the switch cost, the lower the cognitive flexibility.

The switch task was followed by the dual task. The stimulus presentation of the dual task was the same as the switch task. However, stimuli were not presented in a clockwise order, but were presented in only two of the quadrants (either in the letter task or the digit task quadrants, see figure A1b and c). When stimuli were presented in the letter task quadrants, participants were required to respond as fast as possible to the letter (i.e., speeded response). Immediately afterwards (with the same stimulus still present), they had to respond to the digit but could take time to do so. For example, with the letter task they first had to decide as fast as possible whether the letter was lowercase or uppercase. Afterwards, they could take their time to decide whether the digit was

higher or lower than five. Participants first practiced one of the tasks in 24 trials, followed by the actual task of 48 trials. After completing the first task (in this example, the letter task), the participant did the other task (in this example, the digit task, where the speeded response had to be given for the digit and the letter could be evaluated next). The second dual task similarly consisted of 24 practice trials and 48 task trials. Similar to the switch task, the first two trials (i.e., both trials consisting of both a speeded and a slow response) were discarded before analysis. Dual cost was calculated by subtracting the time needed for the non-switch trials of the switch task from the time needed for the speeded response of the dual task. The larger the dual cost, the lower the dual performance.

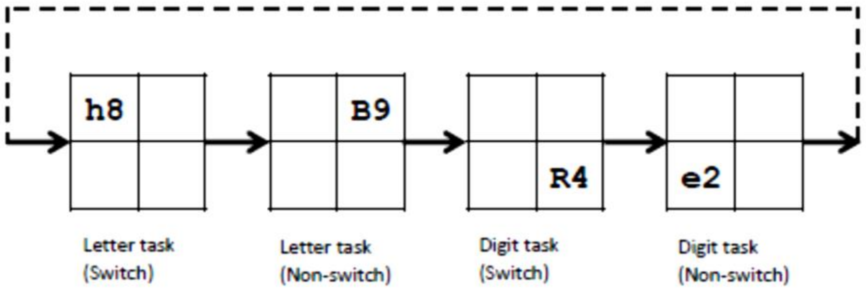

Figure A1a. Illustration of the horizontal version of the switch task. The switch task is based on Rogers & Monsell (1995) [18].

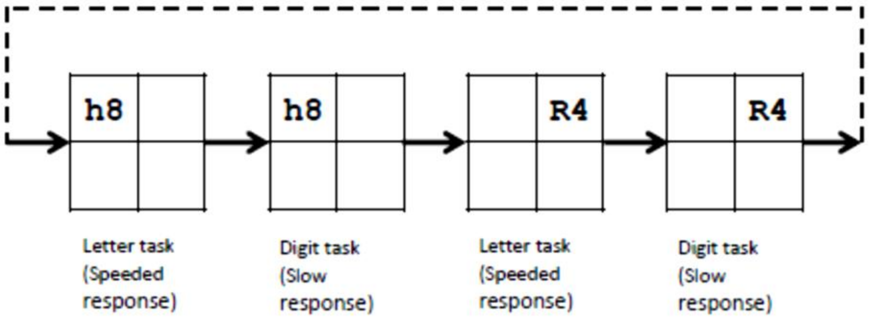

Figure A1b. Illustration of the horizontal version of the dual letter task.

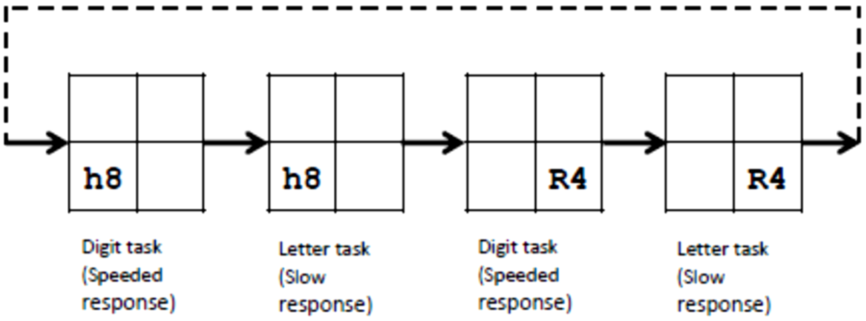

Figure A1c. Illustration of the horizontal version of the dual digit task.

For the switch and the dual tasks, participants were instructed to be as fast and as accurate as possible, such that accuracy was maintained between 91% and 97% correct. An explanation was given of how many items they should have correct to achieve this percentage. Feedback about accuracy was given during each trial immediately below the four quadrants. In the first half of the practice trials, feedback was given to both correct and incorrect answers ("correct" written in green or "wrong" written in red, respectively). In all other trials (including all trials of the actual task) feedback was only given when an error was made. After each block, feedback was given about average accuracy and reaction time. In order to ensure that participants maintained a balance between speed and accuracy, they were instructed to respond faster or to make fewer errors based on their accuracy rate. Participants with accuracy rates above 97% at the end of a block were encouraged to respond more quickly to the stimuli. Similarly, participants performing below an accuracy of 91% were encouraged to make fewer mistakes. When accuracy was between 91% and 97%, participants were instructed to continue in the same way. A reminder of the instructions of which button to press (i.e., left: v = lower case letter and lower than 5; right: N= upper case letter and higher than 5) was present at the bottom part of the screen during the whole task.

Each block started with the presentation of the four quadrants for 2000 ms. In the last 100 ms, a plus mark was presented in the quadrant where the first stimulus would appear in order to prime attention. The stimulus was presented for a maximum of 5000 ms or until a response was given. The interstimulus interval (between response and next stimulus presentation) was 200 ms. However, after an erroneous trial it was extended to 1500 ms during which the word 'Wrong' was presented for 500 ms to indicate error. Note that, when followed by a correct trial, the interstimulus interval immediately returned to 200 ms. For the dual tasks, each stimulus was presented until the participant's second response or until 5000 ms had elapsed. Similar to the switch blocks, after an erroneous dual task trial, the interstimulus interval was extended to 1500 ms during which the word 'Wrong 1st response' and/or 'Wrong 2nd response' was presented for 500 ms to indicate error of a speeded response or second response, respectively.

The stimuli fulfilled three restrictions. First, an equal amount of congruent and incongruent combinations were presented. Second, different characters appeared on each two successive trials. Finally, the same response (i.e., consonant, vowel, odd, or even) could not appear on more than four successive trials.
