# Supplementary material for: The effect of computer-based cognitive flexibility training on recovery of executive function after stroke: rationale, design and methods of the TAPASS study
Source: BMC Neurol. 2015 Aug 20;15:144. doi: 10.1186/s12883-015-0397-y (PMC4545547; doi:10.1186/s12883-015-0397-y)
Supplement: Additional file 4: — Measures at different time-points. (PDF 370 kb) [file 12883_2015_397_MOESM4_ESM.pdf]

Additional file 4

Measures at different time-points

T0<br>Week 1	T1<br>Week 7, 19[a] and 25[a]	T2<br>Week 13	T3<br>Week 17[b]/ 29[a]
	**Primary outcome measures**		
D-Kefs TMT (1-5)		D-Kefs TMT (2,3,4,5)	
ToL (online)	ToL (online)	ToL (online)	ToL (online)
Letter-Number Sequencing		Letter-Number Sequencing	
Fluency		Fluency	
	**Secondary outcome measures**		
Switch +dual task (online)	Switch task (online)	Switch +dual task (online)	Switch task (online)
TMT (online)	TMT (online)	TMT (online)	TMT (online)
DSST (online)	DSST (online)	DSST (online)	DSST (online)
DSC		DSC	
RAVLT		RAVLT	
N-back		N-back	
PASAT		PASAT	
Stop-signal task		Stop-signal task	
Recovery VAS		Recovery VAS	
MRI		MRI	
O-span (online)		O-span (online)	
Corsi (online)	Corsi (online)	Corsi(online)	Corsi (online)
CPM (online)		CPM (online)	
Shipley (online)		Shipley (online)	
DEX (online)	DEX (online)	DEX (online)	DEX (online)
CFQ (online)	CFQ (online)	CFQ (online)	CFQ (online)
USER-P (online)		USER-P (online)	
SF-36 (online)		SF-36 (online)	
IADL (online)		IADL (online)	
HADS (online)		HADS (online)	
CIS-F (online)		CIS-F (online)	
Proxy CFQ (online)		Proxy CFQ (online)	
Proxy DEX (online)		Proxy DEX (online)	
Proxy IADL (online)		Proxy IADL (online)	
	Subjective Training success (online)	Subjective Training success (online)	Subjective Training success (online)
Mouse skills (online)	Mouse skills (online)	Mouse skills (online)	Mouse skills (online)
TICS			
Demographic questions (online)			

Note. [a]Only done by waiting list group; [b]Only done by intervention and active control group; [c]Only done by subgroup of study sample; Shaded cells = done at home

Abbreviation	Measure
D-Kefs TMT	Delis- Kaplan Executive Function System
ToL	Tower of London (Kralen puzzle)
TMT	Trail Making Test (Spoorzoeken)

Abbr. continued	Measure
DSST	Digit Symbol substitution Task
DSC	Digit-Symbol-Coding
RAVLT	Rey Auditory Verbal Learning Task
PASAT	Pased Auditory Serial Addition Test
MRI	Magnetic Resonance Imaging
O-span	Operation span
CPM	Raven Coloured Progressive Matrices
DEX	Dysexecutive Questionnaire
CFQ	Cognitive Failure Questionnaire
USER-P	Utrechtse Schaal voor Evaluatie en Revalidatie- Participatie
SF-36	Short Form Health Survey
IADL	Instrumental activity of daily life scale
HADS	Hospital Anxiety Depression Scale
CIS-F	Checklist Individual Strength- Fatigue subscale
TICS	Telephone Interview Cognitive Status
