# Supplementary material for: The effect of computer-based cognitive flexibility training on recovery of executive function after stroke: rationale, design and methods of the TAPASS study
Source: BMC Neurol. 2015 Aug 20;15:144. doi: 10.1186/s12883-015-0397-y (PMC4545547; doi:10.1186/s12883-015-0397-y)
Supplement: Additional file 5: — Description of the computer tasks. [48–52] (PDF 529 kb) [file 12883_2015_397_MOESM5_ESM.pdf]

Additional file 5

Description of the computer tasks

Tower of London

The online Tower of London (ToL) used in this study has been developed by NeuroTask BV [20]. It is based on the ToL by Culbertson & Zillmer (2005) [16]. Participants were presented with two sets of three rods of decreasing length. In the lower part of the screen, a starting position with three balls was displayed. In the top part of the screen a final position of those three balls was displayed. Participants had to move the balls from the starting position into the final position, using the least amount of steps possible. See figure A2 for an illustration of the task. Ten assignments of increasing complexity were presented. No feedback was given whether the placement of a ball was correct.

Trail Making Test (Spoorzoeken)

The online Trail Making Test (TMT) used in this study is developed by NeuroTask BV. It is based on the TMT by Reitan (1955) [48]. First, a practice trial was presented in which participants had to connect circles with numbers from 1-8 in ascending order by clicking on the circle. Next, participants were presented with a screen with 25 circles including the numbers 1 to 25 (i.e., TMT A). They were required to connect the numbers in ascending order as quickly as possible. After completion, the next practice trial was presented with eight circles, half of them filled with numbers and half of them filled with letters. In this alternating condition (i.e., TMT B), participant had to switch between a letter and a number. The task trials consisted of 25 circles filled with the numbers 1 to 13 and the letters A to L that needed to be connected again in alternating order as quickly as possible. If the participants clicked on a wrong circle, the circle briefly turned red. See figure A3 for an example of what was presented on the screen.

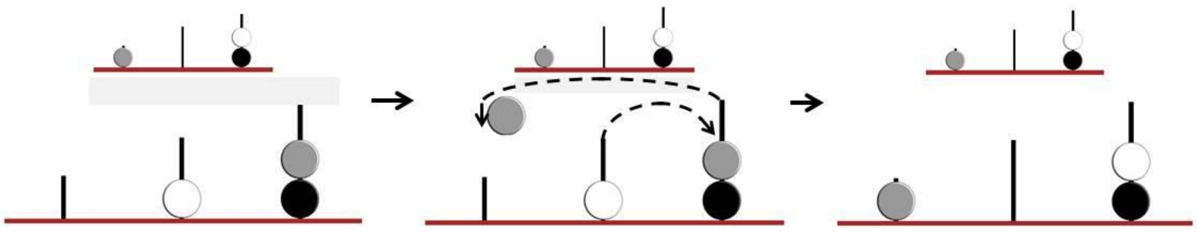

Figure A 2 . Example of the Tower of London (ToL). *Note*: the left represents the starting position; the middle represents how the participant could have moved the balls (represented by the dotted arrows); the right represents the final position.

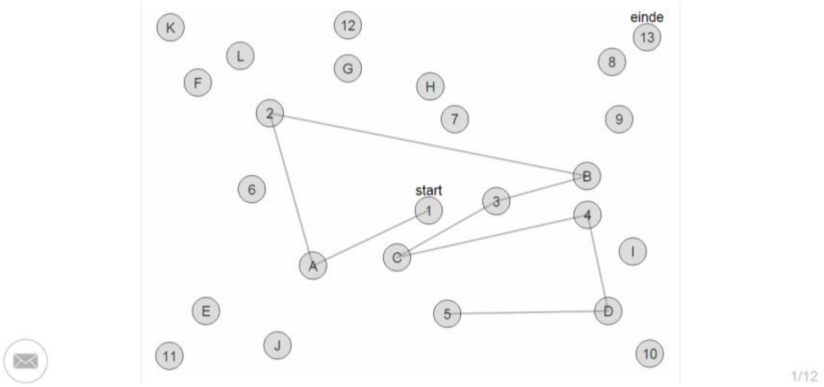

Figure A 3 . Example of the alternating condition of the online Trail Making Test (TMT).

Operation span

The online operation span task was based on the version made by Unsworth, Heitz, Schrock, and Engle (2005) [49]. In this task participants need to remember letters while deciding whether an equation was valid or not. First, both tasks were practiced separately. During the letter practice trials, the participant was presented with a sequence of two or three letters. At the end of a trial, the participant was presented with several letters on the screen and the participant had to click on the letters in the right order of presentation. During the equation practice trials, an equation was presented on the screen. Whenever the participant completed the equation, an answer was presented on the screen and the participant needed to decide whether this was the correct answer or not. In the actual task, the participant saw a letter to remember followed by an equation. Next, a letter was presented on the screen and so on. A sequence of several letters and equations was presented after which the participant was asked again to click on the letters in the same order as they were presented in. As a mental support, the order at which the participants clicked on the letters was represented in boxes next to the letters (see figure A4 for an illustration of this task). Feedback of performance at the equations and letter reproduction was given after the participant had entered the letters and their order of presentation.

N-back

During the n-back task, a white image of an object was presented in the middle of a black screen. The n-back task consisted of three subtasks: a 0-back, a 1-back, and a 2-back task. In the 0-back task, participants needed to press yes (i.e., the 'z' button) if a car was presented on the screen or no (i.e., the '/' button) when any other object was presented on the screen. In the 1-back task, participants needed to press 'z' when the currently presented object was the same as the previous object or press '/' when it was different to the previous object. In the 2-back task, participants needed to press 'z'

when the presented object was the same as the object presented two pictures before the previous one or press '/' when this was not the case (see figure A5. for an illustration of the task). This task was based on the n-back used by de Vries and Geurts (2014) [50]. A detailed description of the task can be found elsewhere [23].

Corsi (Blokkenreeksen)

The Corsi task used in this study was an online version (developed by NeuroTask BV) [20] based on the original Corsi task [51]. An increasing amount of blocks was presented on a screen starting with two blocks. The blocks presented on the screen lit up in a certain sequence (see figure A6. for an illustration of the task). The participant had to reproduce this sequence by clicking on the blocks in the right order. The same amount of blocks but lighting up in a different order was presented twice. If the participant had at least one of the two sequences correct, another block was added to the screen. The task finished when both trials of the same number of blocks were incorrect. The maximum amount of blocks presented on the screen was 11.

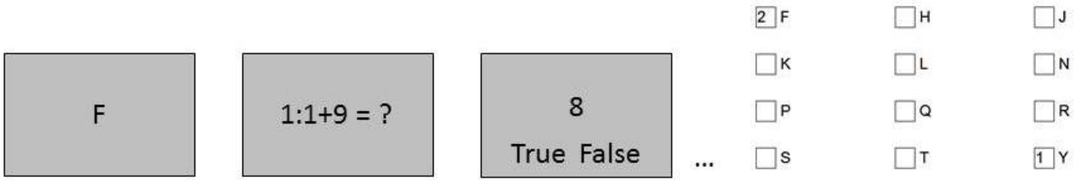

Figure A4. Example of the operation span task.

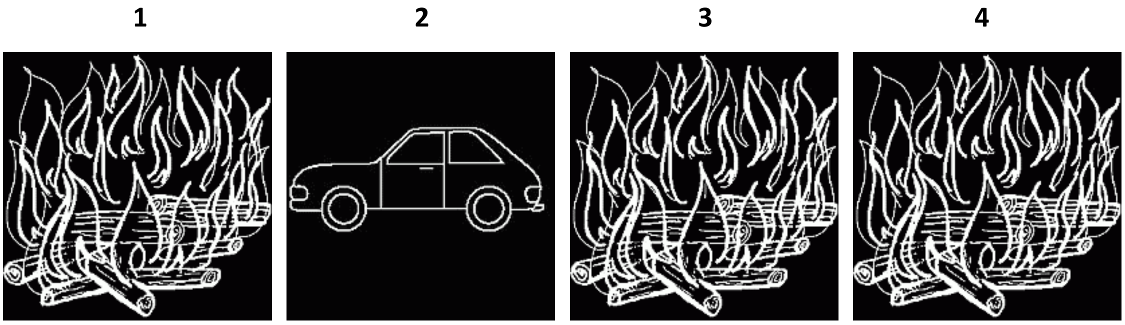

Figure A5. Example of four possible screens in the n-back task. *Note*. Screen two would be a yes in the 0-back task, screen three would be a yes in the 2-back task, and screen four would be a yes in a 1-back task.

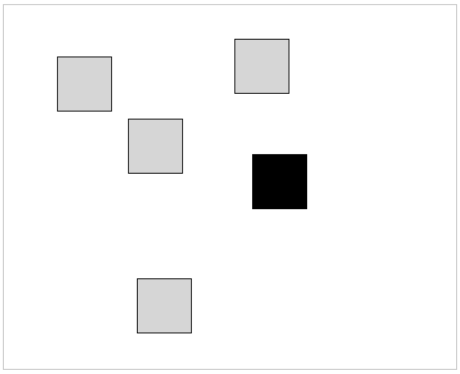

Figure A 6 . Example of the Corsi task.

Mouse skills

Three online tasks were used to determine mouse skills (all developed by NeuroTask BV). In the first task, a 5x5 grid of squares was presented at the middle of the screen. A cross appeared in the top left square. Below the grid, a red square appeared which needed to be placed in the grid at the position of the cross. After correct placement of the square, that square became blue and the cross moved to the next square in the grid. Participant had to complete the grid as fast as possible. See figure A7 for an illustration of the task.

In the second mouse task, a spiral of 25 circles of decreasing sizes was presented on the screen. Participants had to click on the circles in order from the outer circle till the inner circle. After a correct click, the circle turned black. When participants clicked on an incorrect circle, that circle briefly turned red. See figure A8 for an illustration of the task.

The third task was a drag and drop task. Either two circles or two squares were presented opposite to each other somewhere on the screen. Participants had to drag the black object precisely into the white object as fast as possible. See figure A9 for an illustration of the task.

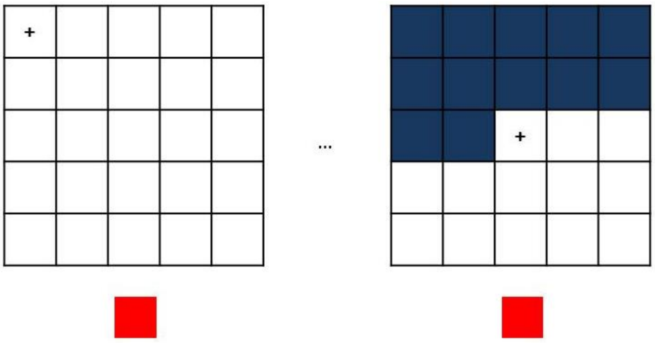

Figure A 7 . Example of the mouse skill task one.

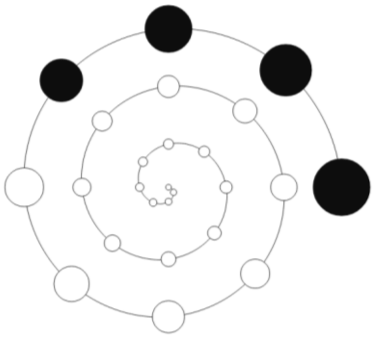

Figure A 8. Example of the mouse skill click task.

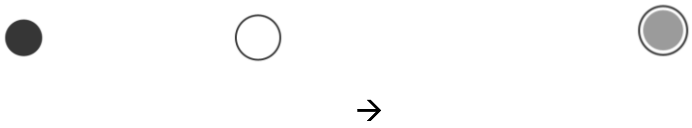

Figure A 9. Example of the mouse skill drag and drop task.

Stop-signal task

In the stop-signal task [52] a green arrow either pointing to the right or to the left was presented on the screen. Participants had to indicate as fast as possible in which direction the arrow was pointing. However, in 20% of the trials the arrow turned red. In these trials, the participant had to inhibit their response such that no response was given (i.e., stop trial). In these stop trials, the interval between the presentation of the green arrow and the arrow turning red differed between trials. The interval started at 300ms and increased with 50ms whenever participant mistakenly pressed a button during a stop trial and decreased by 50ms after a correct withheld response. Stop trials were Participants were stimulated to respond as fast as possible and not to wait till the arrow possibly turned red. See figure A10 for an outline of the task.

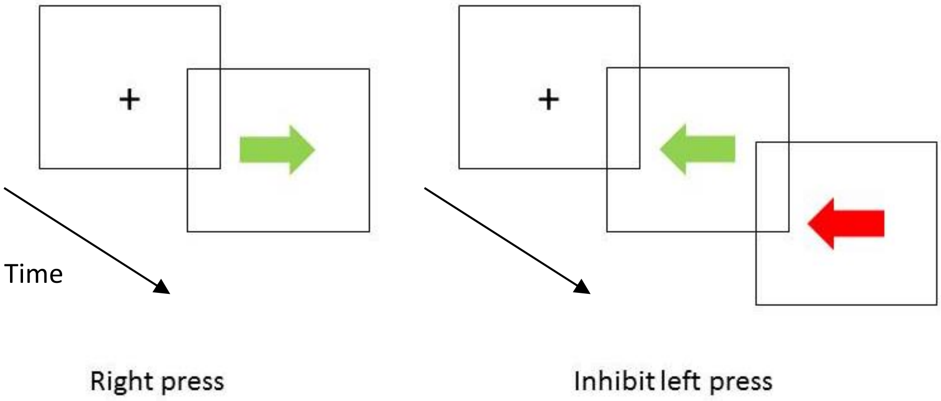

Figure A 10. Example of the stop-signal task. *Note.* The left side of the figure represents a response trial; the right side of the figure represents a stop trial.
